# Obstetric Characteristics and Management of Patients with Postpartum Psychosis in a Tertiary Hospital Setting

**DOI:** 10.1155/2015/386409

**Published:** 2015-05-18

**Authors:** C. E. Shehu, M. A. Yunusa

**Affiliations:** ^1^Department of Obstetrics and Gynaecology, Usmanu Danfodiyo University Teaching Hospital, PMB 2370, Sokoto 840001, Sokoto State, Nigeria; ^2^Department of Psychiatry, Usmanu Danfodiyo University Teaching Hospital, PMB 2370, Sokoto 840001, Sokoto State, Nigeria

## Abstract

*Background*. Postpartum psychosis is the most severe and uncommon form of postnatal affective illness. It constitutes a medical emergency. Acute management emphasizes hospitalization to ensure safety, antipsychotic medication adherence, and treatment of the underlying disorder. *Objective*. The aim of the study was to determine the obstetric characteristics and management of patients with postpartum psychosis in a tertiary centre in North-Western Nigeria. *Methodology*. This was a 10-year retrospective study. Records of the patients diagnosed with postpartum psychosis from January 1st, 2002, to December 31st, 2011, were retrieved and relevant data extracted and analyzed using the SPSS for Windows version 16.0. *Results*. There were 29 cases of postpartum psychosis giving an incidence of 1.1 per 1000 deliveries. The mean age of the patients was 20.6 ± 4 years. Twelve (55%) were primiparae, 16 (72.7%) were unbooked, and 13 (59%) delivered at home. All had vaginal deliveries at term. There were 12 (52.2%) live births, and 11 (47.8%) perinatal deaths and the fetal sex ratio was equal. The most common presentation was talking irrationally. *Conclusion*. There is need for risk factor evaluation for puerperal psychosis during the antenatal period especially in primigravidae and more advocacies to encourage women to book for antenatal care in our environment.

## 1. Introduction

Postpartum psychosis (or puerperal psychosis) is a severe episode of mental illness which begins suddenly in the days or weeks after delivery. It is a dramatic and severe illness with substantial impact in terms of morbidity, marital relationship, and the infant's psychological development [1]. It is the most severe and uncommon form of postnatal affective illness, with rates of 1-2 episodes per 1000 deliveries [2].

The clinical onset is rapid, with symptoms presenting as early as the first 48 to 72 hours postpartum and the majority of episodes developing within the first 2 weeks after delivery. The presenting symptoms are typically depressed or elated mood, disorganized behaviour, mood lability, delusions, and hallucinations [3]. Puerperal hormone shifts [4], obstetrical complications [5], sleep deprivation [6], and increased environmental stress are possible contributing factors to the onset of the illness. Additional risk factors include primiparous patient, family history of psychiatric illness, and personal psychiatric history, particularly a history of mania.

Puerperal psychosis is considered an emergency that necessitates an urgent evaluation, psychiatric referral, and possible hospitalization [7]. Differential diagnoses include bipolar disorder, unipolar major depression, obsessive compulsion symptoms, obsessive compulsion disorders, and schizophrenia [8].

Treatment involves the initiation of acute pharmacotherapy, psychoeducation, supportive therapy, and repeatedly assessing of the patient's function and safety status. The medication options include atypical antipsychotic agents and mood stabilizer or antimanic agents, such as lithium or antiepileptic drugs [9]. Electroconvulsive therapy provides a faster and more complete remission of mood and psychotic symptoms with greater reduction in suicidal ideation and is the mainstream treatment for puerperal psychosis especially in women with intolerable drug side effects [10].

Prognosis is good, especially when symptoms emerge less than one month after delivery [11]; however, they are at risk of developing further puerperal and nonpuerperal episodes of bipolar affective disorder [12].

The aim of this study was to review the obstetric characteristics and management of postpartum psychosis in a tertiary hospital in Sokoto, North-Western Nigeria.

## 2. Methods

This was a 10-year retrospective descriptive study. Case notes of patients diagnosed with postpartum psychosis, using the ICD 10 diagnostic criteria, from January 1st, 2002, to December 31st, 2011, at Usmanu Danfodiyo University Teaching Hospital, Sokoto, were retrieved manually from the health records department. Data relating to age, parity, presentation, risk factors, maternal and foetal morbidity, and mortality were extracted and analyzed using the SPSS for Windows version 16.0. Ethical approval for the study was from the Hospital Ethics Committee.

## 3. Results

Twenty-five thousand, nine hundred and fifty deliveries occurred in the ten years under review with 29 cases of postpartum psychosis giving an incidence of 1.1 per 1000 deliveries. However, 22 case notes were available for analysis giving a retrieval rate of 75.9%.

The ages of the patients ranged between 16 and 32 years with a mean age of 20.6 ± 4 years (Table 1).

Majority, 20 (91.0%), of the patients were Hausa while other tribes accounted for 2 (9.0%) patients. All the patients were married. Fifteen (68.2%) were in a monogamous setting and 3 (13.6%) were in a polygamous setting while, in 4 (18.2%) patients, the marriage setting was not declared. All the women were housewives and were not gainfully employed and only one woman had formal education up to secondary level (Table 2).

Thirteen (59.1%) were primiparous and 8 (36.4%) were multiparous while 1 (4.5%) was a grand multipara. Sixteen (72.7%) were unbooked and 5 (22.7%) were booked but the booking status of one patient (4.6%) was not stated. Thirteen (59.0%) had unsupervised home delivery and the other 9 (41.0%) delivered in a hospital setting. Majority, 21 (95.4%), had spontaneous vaginal delivery and all the deliveries were at term. There were 12 (52.2%) live births, 8 (34.8%) still births, and 3 (13.0%) early neonatal deaths in the group. One of the women had a set of still born female twins. There were 9 (39.1%) male and 10 (43.5%) female neonates with 4 (17.4%) neonates' sex not stated (Table 3).

The most common clinical presentation was talking irrationally in 86.4% of patients. Other forms of presentation were fever (22.7%), refusal to eat (18.2%), mutism (4.5%), refusal to breastfeed (4.5%), suicidal attempt (4.5%), and infanticidal ideation (4.5%). Eight patients (36.4%) presented to the hospital within 72 hours of onset of symptoms and another 8 presented within the first 2 weeks while the rest presented after 2 weeks of onset of the symptoms.

Comorbidities associated with the illness were malaria (22.7%), puerperal sepsis (22.7%), intra-/postpartum eclampsia (22.7%), and severe anaemia (9.2%). Five patients (22.7%) had no comorbidity.

**Figure 1:**
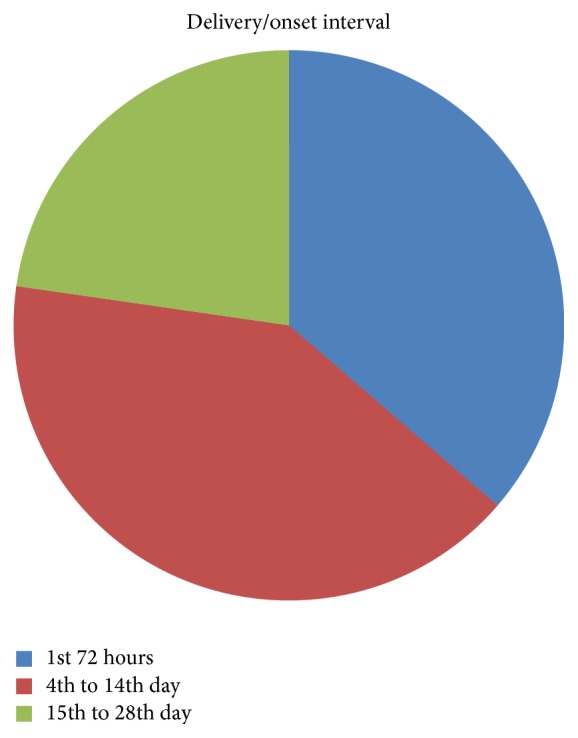


The interval between delivery and onset of postpartum psychosis was 72 hours in 8 (36.3%) patients, within 14 days postpartum in 9 (40.9%) and within the first 4 weeks in the remaining 5 (22.7%) patients (Figure 1).

None of the patients had a prior history of psychiatric illness while 2 (9.1%) had suffered from postpartum psychosis in a previous delivery. Only one woman had a history of chewing* Cola nitida* excessively.

The mainstay of treatment was by the use of pharmacotherapy (imipramine, haloperidol, and benzhexol) in all the patients. However, one patient had electroconvulsive therapy for lack of adequate response to pharmacotherapy. Eleven (50%) patients were discharged home after treatment while the other 11 (50%) were removed from the hospital by their relatives before treatment was completed. There was no maternal mortality recorded among the women.

## 4. Discussion

This was a retrospective study of case files of women who were diagnosed with postpartum psychosis, using the ICD 10 diagnostic criteria, Sokoto, North-Western Nigeria. Of the 29 cases recorded, 22 case files were available for analysis. This is one of the limitations of retrospective reviews especially in developing countries where record keeping may be suboptimal.

The incidence of 1.1 per 1000 deliveries found in this study is comparable to that found in other studies [2, 3, 11, 13]; however it was lower than the incidence in Sagamu, South-Western Nigeria [14].

The ages of the patients' ranged between 16 and 32 years with a mean age of 20.63 ± 4 years. This finding differs from that of a study in Sweden which found that older maternal age was associated with increased risk of first hospital admission from postpartum psychosis among first-time mothers [15]. This difference may be because the average age at first birth in Nigeria is less than 19 years [16] and primiparity is reported as a risk factor for postpartum psychosis [1, 5, 6, 14].

All the patients were married with majority in a monogamous setting. All were housewives and were not gainfully employed with only one woman who had formal education up to secondary level. These findings agree with that of studies in Pakistan [17] and Sweden [18] where it was shown that women living in the poorest neighbourhoods exhibited a significantly higher risk of postpartum psychosis than women living in the richest neighbourhoods.

Majority (72.7%) of the women did not have the benefit of antenatal care or delivery by skilled birth attendants either at home or in a hospital setting. This may explain the high perinatal deaths (47.8%) recorded among them as it has been shown that providing skilled care at birth not only reduces maternal mortality but has been found to also reduce perinatal mortality [19]. Furthermore, high perinatal mortality rates are prevalent in our study area even after adjusting for confounders [20]. The infants in this study group were nursed separately at the Special Care Baby Unit of the hospital till their mothers recovered from the acute phase of the illness.

Talking irrationally was the most common form of presentation occurring in 86.4% of patients. This finding was different from what was reported in most literature [2, 3, 11] where the symptoms were depressed or elated mood, disorganized behaviour, delusions, and hallucinations. However, this difference could be because of cultural variations. In Nigeria, child birth is highly celebrated and the new mother is usually expected to be in high spirits especially after a successful delivery. Thus, early symptoms of the illness may go unnoticed and the patient is often brought in with severe psychiatric presentations. Majority of the patients in this study presented after 72 hours of onset of symptoms. This pattern of presentation was a similar finding in an earlier study [5].

The physical comorbidities associated with postpartum psychosis in this study were mostly malaria, puerperal sepsis, and lastly eclampsia. This was in slight contrast to the finding in Tanzania [21] where anaemia topped the list of physical comorbidity followed by infections and preeclampsia. Our findings were not surprising as many of the patients were prone to the morbidities because they had not received antenatal care or delivery by skilled birth attendants.

Postpartum psychosis occurred within the first two weeks of delivery in majority (77.2%) of the patients in this study. However, this finding did not agree with findings from Abeokuta [22] where the illness occurred after this period.

Only two women in this study had a prior history of postpartum psychosis and none had a prior history of psychiatric illness. None also volunteered a family history of psychiatric illness. This is not the usual finding in the literature [5, 14, 16, 20, 23] where the risk of postpartum psychosis has been shown to be increased in families with a history of bipolar disorder. However, a lot of stigmas are attached to psychiatric illnesses in Africa as a whole; thus most people will not willingly volunteer such information. Also, one woman had a history of chewing* Cola nitida* [24] excessively but this has not been listed as a risk factor to the illness in any study yet.

The mainstay of treatment in this study was pharmacotherapy (imipramine, haloperidol, and benzhexol) in all the patients. However, only one patient had electroconvulsive therapy (ECT) for lack of adequate response to pharmacotherapy. ECT becomes the therapeutic option where risk of infanticide, suicide, or refusal to take drugs is high. Some studies advocate the use of ECT as first line treatment in selected cases [25] while others recommend pharmacotherapy [26].

About 50% of the patients were removed from the hospital by their relatives before treatment was completed. This is a common occurrence in the management of psychiatric illnesses in Nigeria where relatives prefer treatment by religious or traditional healers above orthodox medicine [27]. It is noteworthy that there was no maternal mortality recorded among the women who completed treatment and were discharged home.

## 5. Conclusion

The incidence of postpartum psychosis was 1.1 per 1000 deliveries in our centre. Primiparity appeared to be a risk factor and the illness occurred mainly in unbooked patients who also had unsupervised home deliveries. The most common mode of presentation was irrational talks.

There is need for risk factor evaluation for puerperal psychosis during the antenatal period especially in primigravidae and more advocacies to encourage women to book for antenatal care in our environment.

## Figures and Tables

**Table 1:** Age distribution of the patients.

Age	Number	(%)
15–19	12	54.6
20–24	4	18.2
25–29	5	22.7
30–34	1	4.5
Total	**22**	**100**

**Table 2:** Sociodemographic characteristics of the patients.

Variable	Number	(%)
Ethnic group		
Hausa	20	91.0
Fulani	1	4.5
Igbo	1	4.5
Total	**22**	**100**
Religion		
Islam	21	95.5
Christianity	1	4.5
Total	**22**	**100**
Type of marriage		
Monogamy	15	68.2
Polygamy	3	13.6
Not stated	4	18.2
Total	**251**	**100**
Educational status		
No formal education	21	95.5
Secondary	1	4.5
Total	**22**	**100**

**Table 3:** Obstetric characteristics of the patients.

Obstetric characteristic	Number	(%)
Parity		
Primipara	13	59.1
Multipara	8	36.4
Grand multipara	1	4.5
Total	**22**	**100**
Booking status		
Booked	5	22.7
Unbooked	16	72.7
Not stated	1	4.6
Total	**22**	**100**
Place of delivery		
Hospital	13	59
Home	9	41
Total	**22**	**100**
Type of delivery		
Spontaneous vaginal delivery	21	95.5
Assisted (vacuum) delivery	1	4.5
Total	**22**	**100**
Neonatal outcome		
Live births	12	52.2
Still births	8	34.8
Early neonatal deaths	3	13.0
Total	**23**	**100**
Neonate's sex		
Female	10	39.1
Male	9	43.5
Not stated	4	17.4
Total	**23**	**100**
